# Accessing sub-national cholera epidemiological data for Nigeria and the Democratic Republic of Congo during the seventh pandemic

**DOI:** 10.1186/s12879-022-07266-w

**Published:** 2022-03-26

**Authors:** Gina E. C. Charnley, Ilan Kelman, Katy A. M. Gaythorpe, Kris A. Murray

**Affiliations:** 1 School of Public Health, Imperial College London, Norfolk Place, London, W2 1PG UK; 2 Institute for Global Health & Institute for Risk and Disaster Reduction, University College London, London, WC1E 6BT UK; 3 University of Agder, Kristiansand, Norway; 4 Centre for Global Infectious Disease Analysis, School of Public Health, Imperial College London, London, W2 1PG UK; 5 MRC Unit the Gambia at London School of Hygiene and Tropical Medicine, Atlantic Boulevard, Fajara, The Gambia

**Keywords:** Public Health, Epidemiology, Cholera, *Vibrio cholerae*, Nigeria, Democratic Republic of Congo

## Abstract

**Background:**

*Vibrio cholerae* is a water-borne pathogen with a global burden estimate at 1.4 to 4.0 million annual cases. Over 94% of these cases are reported in Africa and more research is needed to understand cholera dynamics in the region. Cholera data are lacking, mainly due to reporting issues, creating barriers for widespread research on cholera epidemiology and management in Africa.

**Main body:**

Here, we present datasets that were created to help address this gap, collating freely available sub-national cholera data for Nigeria and the Democratic Republic of Congo. The data were collated from a variety of English and French publicly available sources, including the World Health Organization, PubMed, UNICEF, EM-DAT, the Nigerian CDC and peer-reviewed literature. These data include information on cases, deaths, age, gender, oral cholera vaccination, risk factors and interventions.

**Conclusion:**

These datasets can facilitate qualitative, quantitative and mixed methods research in these two high burden countries to assist in public health planning. The data can be used in collaboration with organisations in the two countries, which have also collected data or undertaking research. By making the data and methods available, we aim to encourage their use and further data collection and compilation to help improve the data gaps for cholera in Africa.

## Background

Communicable diseases still result in a large proportion of global mortality despite advances in sanitation, hygiene and vaccination, with diarrheal diseases categorised as the eighth leading cause of death worldwide [1]. For instance, *Vibrio cholerae* is a water-borne pathogen which contributes to diarrhoeal disease mortality, especially in low and middle-income countries. It is considered a disease of inequity, primarily because of its association with poverty, poor access to water, sanitation and hygiene (WASH) and inability to access healthcare [2]. The global burden of cholera is estimated at 1.4–4.0 million annual cases and 21,000 to 143,000 annual deaths [3]. Despite over 94% of World Health Organization (WHO) reported cholera cases occurring in Africa and high mortality rates, cholera research is heavily focussed on South America, the Indian subcontinent and more recently Haiti [4]. More research and published studies on cholera in Africa are needed to better understand cholera dynamics there. Additional work on the risk factors for cholera outbreak occurrence could help inform effective public health planning and significantly reduce cholera incidence and outbreaks on the continent.

A barrier to this research is a chronic and persistent lack of data to evaluate, especially sub-nationally, preventing routine risk assessments and quantitative research including cholera dynamic modelling. Freely and publicly available cholera data are very rarely sub-national and only provide case and death numbers on a national annual level [5]. There are also issues with under-reporting of cholera cases and deaths, with the WHO’s most optimistic estimates stating that ~ 10% of cases are captured in the data [6]. Issues in reporting occur due to a variety of reasons including a spectrum of transmission dynamics, requiring several surveillance resources to capture them all. Lulls in cases reducing focus on cholera and new outbreaks shift attention and funding. There are also barriers to accessing healthcare and disincentives to report including fears for safety and restrictions on travel and trade [7, 8].

To help bridge these cholera data gaps, we are making data used in previous research publicly available and providing information on the data collection methods used to improve transparency and quality control. The data are sub-national and where possible age and sex-disaggregated data for two high burden African countries: Nigeria and the Democratic Republic of Congo (DRC). Both countries have suffered from large outbreaks during the seventh pandemic which emerged in Africa in the 1970s [2] and currently have the second (Nigeria) and third (DRC) highest number of reported cholera cases per year in Africa [9]. These outbreaks have been worsened by violent conflicts, poor access to WASH and poverty [10, 11]. The data presented here can help to encourage both qualitative and quantitative cholera research in Nigeria and the DRC, to inform public health and enhance the understanding of cholera dynamics. The data were extracted from publicly available sources and therefore are freely available to use. Despite this, we hope these data will be used in collaborations with those working on cholera in Nigeria and the DRC in health and academic institutions.

## Construction and content

A range of publicly available sources was used to collate, update and curate the datasets. All available data were included from these sources on case and death numbers for cholera outbreaks from the WHO’s disease outbreak news [12], ProMED [13] (which included ReliefWeb), WHO’s regional office for Africa weekly outbreak and emergencies [14], UNICEF cholera platform [15], EM-DAT [16] and the Nigeria Centre for Disease Control (NCDC) [17]. A literature search using MEDLINE, Embase, Global Health and Google Scholar (with snowballing of reference lists) was also completed, and data were included from studies which met the inclusion and exclusion criteria (Table 1). A full list of the references from the literature search is shown in the data files and included 18 studies from Nigeria and seven from the DRC. Where search terms were needed, they included *Vibrio cholerae* and “cholera” for ProMED and additionally “Nigeria”, “Democratic Republic of Congo” and “DRC”. No temporal limits were set, as these could exclude important articles. For ProMED both English and French outbreak reports were reviewed and incorporated into the datasets.

**Table 1:** Eligibility criteria for the literature search; literature which met the criteria are shown in the data files

Inclusion	
Population	Any local population/community impacted by a cholera outbreak in Nigeria or the Democratic Republic of Congo
Intervention	Any investigation carried out to quantify and understand cholera cases/deaths and risk factors
Comparator	Anyone in the affected population or community which did not become infected with cholera
Outcomes	The outcome is to understand epidemiological features of cholera outbreaks in Nigeria and the Democratic Republic of Congo
Study type	Retrospective observational reports/studies including cross-sectional, case–control and cohort studies
Exclusion	
Studies which investigate a diarrhoeal disease outbreak with no specific mention of cholera	
Serological surveys evaluating antibody levels in the population and not a specific outbreak with active cases	
Review papers, as only primary sources were used	
Publications looking at public health and cholera prevention more generally and not in relation to a specific response	
Non-English or non-French abstracts and full-texts, due to linguistic constraints	
Multiple data points where the information reported was the same	

A data charting form was used to store the data using Microsoft Excel and was organised by Entry ID. Entry ID was formatted as country, year, month, day and an additional number to account for entries for the same country and day (e.g., NGA71-3-1-1, Nigeria-1970-March-1st-entry no. 1). Additional columns included date (day month), year, state/province, local government area/territory, health zone, cases, deaths, confirmed, hospitalised, fatality rate (%), male or female, age, oral cholera vaccine (OCV) delivered, population (as a national annual figure), cases/100,000, deaths/100,000 (calculated using WorldBank population data [18]) and source. Information was also collected on risk factors and interventions stated by the sources and an additional twelve columns were provided to track these, including displacement, socio-cultural factors, food/water, environment, rural or urban, sanitation and hygiene, education, occupation, household/setting, conflict (Y meaning yes; no mention of conflict was left blank), intervention and aid.

**Fig. 1:**
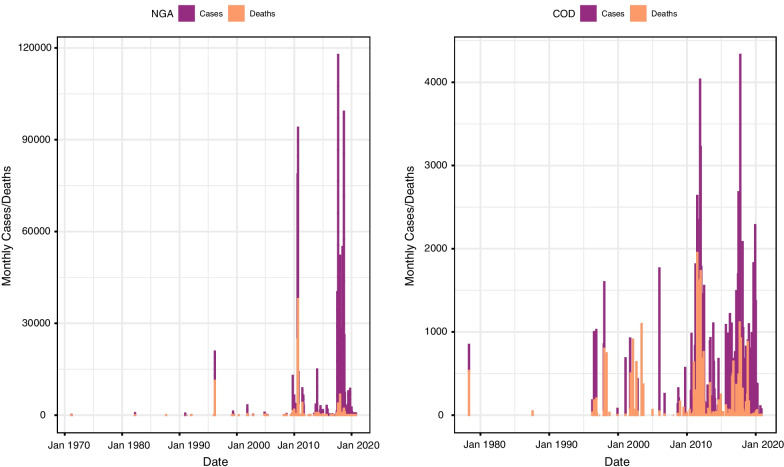
Temporal trends of cholera cases and deaths for the full datasets for Nigeria (NGA) and the Democratic Republic of Congo (COD)

**Fig. 2:**
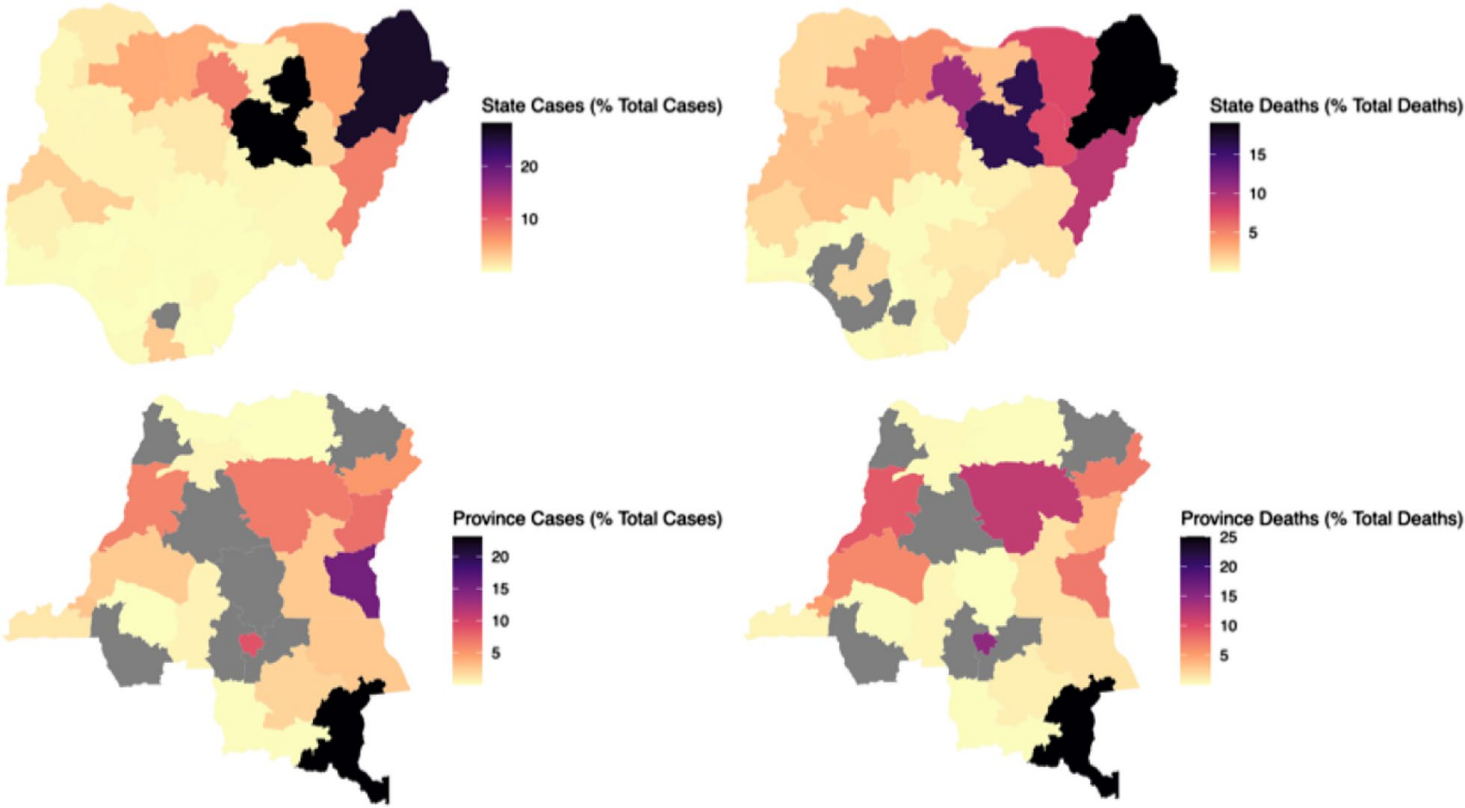
Spatial distribution of cholera cases and deaths to administrative level 1 for the full datasets for Nigeria (top) and the Democratic Republic of Congo (bottom). The shapefiles are under Creative Commons Attribution licenses, more specifically CC BY-IGO for the DRC [24] and CC-BY 4.0 for Nigeria [25]. They are free to share and or adapt for any purpose

Data are provided on the finest spatial scale that the source allowed, which in some entries is to administrative level 3 (heath zones in the DRC, while Nigeria does not have this level). For the DRC, province names (administrative level 1) and borders have changed, most recently in 2015. If the pre-2015 province name or border was used by a source, then the post-2015 name and border were identified and used. These were delineated using a working paper developed by the Claremont Graduate Institute [19]. Data spanned from 1971 to 2020 for Nigeria and from 1978 to 2020 for DRC. Temporal and spatial summaries of these data are shown in Figs. 1 and 2. These were the maximum date ranges in which data were found for the two countries. The data were recorded by the date of the report, which was on a daily temporal scale (DD-MM-YY). Where data were not available for a certain column, this was left blank and each row represents a case or death entry for a specific location. At the time of this manuscript’s submission, the maximum number of data entry points found was 1334 for Nigeria and 607 for the DRC.

As multiple sources were used, in some instances, the same outbreak was reported by more than one source. In this case, all reports were entered into the data until the end of the data entry process. If the entries were the same, then the subsequent reports were deleted but the source column held all the sources reviewed. If entries were different, then an average was taken from all sources that reported on the outbreak and each reference was provided in the source column. By using an average of multiple sources our intention was to create a more accurate measure of cholera cases and deaths. In cases where the reports were the same, this allowed an element of validation of the data.

The datasets were then reviewed, and initially cleaned manually, after the data collection was finished. The aim of this was to streamline terminology in the risk factors and interventions used, along with differences in the way demographic changes were reported. Doing this manually helped to identify errors in the data entry process and, for individual records, to be checked against the sources again if needed.

## Utility and discussion

### Intended uses/advantages of the datasets

The data presented here will be useful for academic, private and public institutions looking to carry out qualitative, quantitative and mixed-method research into cholera dynamics in Nigeria and the DRC. The freely available sources of the data and the anonymisation avoid issues of using more sensitive/private datasets such as lengthy ethical approval processes. The immediate availability of the data means they can be used in creating hypotheses and for initial analyses, while more sensitive data are obtained and data privacy agreements are signed. We have used the data in our own research investigating the impacts of conflict on cholera in Nigeria and the DRC and a pre-print of this work is available [20]. The nature of the data allowed for the continuation of the cholera research in this project, while connections and contacts were built in the target countries.

### Limitations of the datasets

When curating the datasets, cases and deaths were treated as suspected, unless stated otherwise, which were inputted into a separate column (labelled ‘confirmed’). Due to the differences in cholera case definitions [21, 22], this approach may have created errors in the data. For example, only severe cholera cases were reported. No information on cholera strain was given, as sources which specified strain were limited, and the only strains stated were *Vibrio cholerae* O1 and *Vibrio cholerae* O1 El Tor, as the cause of outbreaks.

Entry ID represents each new report of cases and/or deaths from the sources. Although this provides valuable information on those infected, outbreak investigation would still be needed to understand the full evolution of the outbreaks which were reported here. It is difficult from individual reports to understand transmission dynamics and full outbreak investigations. Despite this, the datasets presented here help to provide a more accurate snapshot of cholera burden in Nigeria and the DRC, compared to other publicly available datasets.

**Fig. 3:**
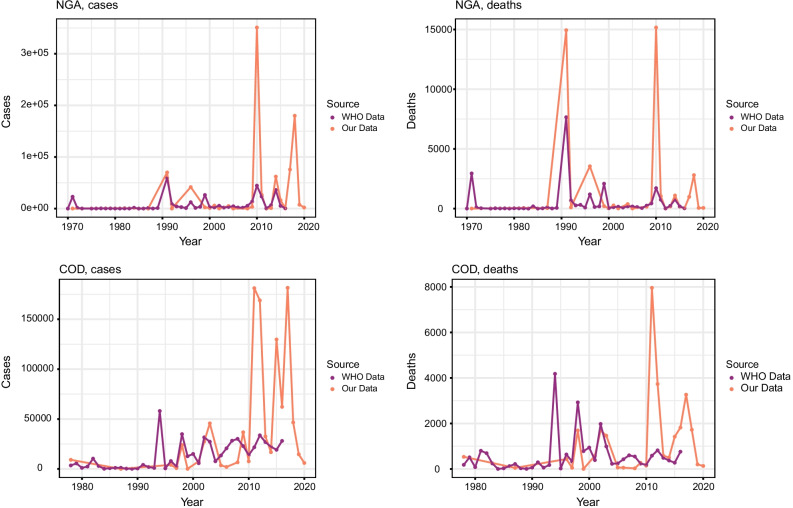
Comparison of cholera cases and deaths for the full datasets presented here compared to the WHO’s Global Health Observatory for Nigeria (NGA) and the DRC (COD) [23]

Under-reporting is a systemic issue for cholera data and a limitation of the data presented here. During cholera outbreaks, healthcare and surveillance systems are often overwhelmed, meaning cases can be missed. To understand the extent to which this issue impacted our data, we compared our data to the WHO Global Health Observatory Data [23]. Our data showed similar trends with more cases and deaths reported in our datasets, as expected given the under-reporting to WHO (Fig. 3). Calculated correlation coefficients for the two datasets showed that only Nigeria had a statistically significant correlation (p ≤ 0.05). This is potentially due to the additional data source (NCDC) for Nigeria, meaning cholera was more accurately captured in the Nigeria datasets. We also compared our data for Nigeria and the DRC with data we obtained from the Nigeria Centre for Disease Control and Johns Hopkins Bloomberg School of Public Health. Our data correlated well with these private sources, although details of this cannot be given here due to data privacy and data sharing agreements.

### Future work and developments

We hope that sharing both our data and methods will help to facilitate and encourage others working in cholera research to do the same, to create a more accurate consensus of global cholera burden. We hope this process can be repeated for other countries and diseases, especially neglected tropical diseases which are often chronically understudied and lack funding for surveillance. Combining data from several sources can create global and internationally supported datasets for both public health and scientific research. The methodology used here allows for a dynamic data entry process, that can be easily updated as new reports and sources become available, while still allowing for the original source of the data to be easily tracked to increase transparency and reduce error.

## Conclusions

We expect the datasets presented here to be used in a variety of cholera research methods and areas in these two high burden countries to assist in public health planning. The data should be used in collaboration with academic, public and private organisations in the host countries, generating global partnerships. By making the data and methods available, we aim to encourage further data collection and compilation which can be published, to help further bridge the data gaps for cholera in Africa.

## Data Availability

The datasets generated during this current study are available in a Github repository, available at: https://github.com/GinaCharnley/cholera_data_drc_nga. The repository includes the datasets that were last updated in February 2021. The repository includes the full data charting form, along with two cleaned datasets that were used to make summary figures of the data. The full data charting form also includes hyperlinks to the primary sources. These include cleaned cholera cases and deaths files and cleaned datasets of the risk factors, interventions and demographic differences. These are available in CSV format that can be easy downloaded and imported into a variety of software programmes. The R code used in the cleaning process and for the summary figures, along with any changes made to the dataset, are also included in the repository.

## Abbreviations

WASH: Water, Sanitation and Hygiene
WHO: World Health Organization
DRC: Democratic Republic of Congo
NCDC: Nigeria Centre for Disease Control
OCV: Oral cholera vaccine
